# Your Resting Brain CAREs about Your Risky Behavior

**DOI:** 10.1371/journal.pone.0012296

**Published:** 2010-08-19

**Authors:** Christine L. Cox, Kristin Gotimer, Amy K. Roy, F. Xavier Castellanos, Michael P. Milham, Clare Kelly

**Affiliations:** 1 Phyllis Green and Randolph Cōwen Institute for Pediatric Neuroscience at the New York University Child Study Center, New York University Langone Medical Center, New York, New York, United States of America; 2 Nathan Kline Institute for Psychiatric Research, Orangeburg, New York, United States of America; CNRS, France

## Abstract

**Background:**

Research on the neural correlates of risk-related behaviors and personality traits has provided insight into mechanisms underlying both normal and pathological decision-making. Task-based neuroimaging studies implicate a distributed network of brain regions in risky decision-making. What remains to be understood are the interactions between these regions and their relation to individual differences in personality variables associated with real-world risk-taking.

**Methodology/Principal Findings:**

We employed resting state functional magnetic resonance imaging (R-fMRI) and resting state functional connectivity (RSFC) methods to investigate differences in the brain's intrinsic functional architecture associated with beliefs about the consequences of risky behavior. We obtained an individual measure of expected benefit from engaging in risky behavior, indicating a risk seeking or risk-averse personality, for each of 21 participants from whom we also collected a series of R-fMRI scans. The expected benefit scores were entered in statistical models assessing the RSFC of brain regions consistently implicated in both the evaluation of risk and reward, and cognitive control (i.e., orbitofrontal cortex, nucleus accumbens, lateral prefrontal cortex, dorsal anterior cingulate). We specifically focused on significant brain-behavior relationships that were stable across R-fMRI scans collected one year apart. Two stable expected benefit-RSFC relationships were observed: decreased expected benefit (increased risk-aversion) was associated with 1) stronger positive functional connectivity between right inferior frontal gyrus (IFG) and right insula, and 2) weaker negative functional connectivity between left nucleus accumbens and right parieto-occipital cortex.

**Conclusions/Significance:**

Task-based activation in the IFG and insula has been associated with risk-aversion, while activation in the nucleus accumbens and parietal cortex has been associated with both risk seeking and risk-averse tendencies. Our results suggest that individual differences in attitudes toward risk-taking are reflected in the brain's functional architecture and may have implications for engaging in real-world risky behaviors.

## Introduction

Risk seeking and risk-avoidance play a central role in both normal and pathological decision-making. As such, characterizing the neural correlates of these traits and behaviors is a central focus of cognitive neuroscience and psychiatric research. Individuals with anxiety disorders, and those with high but not pathological levels of anxiety, exhibit high levels of risk-aversion [1]. On the other hand, disorders such as pathological gambling and alcohol and drug abuse are associated with increased risk-taking behavior [2]–[4]. Individuals with substance abuse and anxiety disorders also exhibit structural and functional abnormalities in brain regions critical for adaptive decision-making [5]–[7].

A distributed network of brain regions has been implicated in task-based neuroimaging studies of risky decision-making, including orbitofrontal cortex (OFC), ventral striatum (nucleus accumbens), insula, anterior cingulate, and lateral frontal and parietal cortices [8], [9]. Alterations in the function of these regions have been linked to changes in risky behavior. Patients with OFC lesions exhibit exaggerated risk-taking behavior on gambling tasks compared to nonfrontal lesion patients and healthy controls [10]. In healthy adults, temporary disruption of right dorsolateral prefrontal cortex (DLPFC) function via repetitive transcranial magnetic stimulation has been associated with increased risky decision-making during gambling tasks [11]. Conversely, temporary enhancement of DLPFC function via transcranial direct current stimulation has been associated with increased risk-aversion [12].

Individual differences in risk-related personality factors have also been linked to individual differences in activation in several of the aforementioned brain regions. Higher levels of behavioral risk-aversion and harm-avoidance have been associated with increased activation in the inferior frontal gyrus (IFG) and insula [13], [14] (but see [15]). Conversely, increased risk seeking preferences have been associated with increased OFC activation [15], [16]. In addition, Galvan and colleagues [17] reported that individuals' likelihood of engaging in risky behavior, as well as their expectations of positive consequences from risk-taking, were positively correlated with increased nucleus accumbens activation during reward anticipation. A recent study showed that the distinct developmental trajectories of brain networks involved in reward processing (i.e., ventromedial prefrontal cortex and ventral striatum) and cognitive control (i.e., dorsal anterior cingulate and lateral prefrontal cortices) are associated with increased risky decision-making in adolescence [18]. In the same study, individual differences in the propensity to take risks modulated activation in these networks across development.

Together, existing findings suggest that interactions among brain regions supporting the evaluation of risk and reward (e.g., OFC, nucleus accumbens [19]–[22]), and those supporting cognitive control over thoughts and behavior (e.g., dorsal anterior cingulate and lateral prefrontal cortices [23]–[25]) contribute to adaptive decision-making. These interactions may contribute to a bias toward either “risky” or “safe” behavior. A central challenge for studies of risky behavior is the development of behavioral paradigms that concurrently tap these diverse neural circuits and assess risk-taking or risk-averse tendencies in an ecologically valid manner. Furthermore, it is important to consider how individual differences in trait, as opposed to state, personality variables (e.g., risk seeking, risk-aversion) may contribute to differences in the activity of and interactions between these networks.

Resting state functional magnetic resonance imaging (R-fMRI) approaches characterize functional brain networks while avoiding some of the constraints of task-based approaches [26]–[29]. Networks identified using resting state functional connectivity (RSFC) analyses closely correspond to networks of co-activated brain regions observed when individuals carry out a variety of tasks, suggesting that they are intrinsic representations of the brain's functional repertoire [30], [31]. In addition, individual differences in trait measures (e.g., social competence) have been shown to predict individual differences in RSFC between brain regions [32]–[34]. This supports the utility of these methods for investigating enduring brain-behavior relationships.

Here, we employed R-fMRI approaches to identify differences in RSFC predicted by individual differences in a trait measure of risk-related cognitions. Specifically, we investigated whether patterns of RSFC in brain regions previously implicated in risky decision-making were related to individuals' beliefs about the consequences of engaging in risky behaviors. Furthermore, we sought to identify brain-behavior relationships that were stable across time (∼1 year). In order to do this, we administered the Cognitive Appraisal of Risky Events (CARE) questionnaire [35] to participants from whom we also acquired a set of R-fMRI scans. The CARE measures participants' expected benefit from engaging in real-world risky behaviors such that high and low scores can be considered indicative of a more risk seeking or risk-averse personality, respectively. We examined relationships between expected benefit scores and RSFC within risk-related circuits. We then determined which relationships were consistent across time, indicating a stable and enduring association between personality trait and intrinsic brain connectivity.

We predicted that expected benefit scores would be negatively related to RSFC between brain regions associated with *cognitive control and risk-aversion* (e.g., lateral prefrontal cortex, dorsal anterior cingulate, insula). Namely, increased expected benefit, or increased risk seeking, would be associated with weaker RSFC between these regions. Similarly, decreased expected benefit, or increased risk-aversion, would be associated with stronger RSFC between these regions. For brain regions associated with *reward and risk seeking* (e.g., nucleus accumbens, OFC), we predicted expected benefit scores to be positively related to RSFC. Namely, increased expected benefit (risk seeking) would be associated with stronger RSFC between these regions, while decreased expected benefit (risk-aversion) would be associated with weaker RSFC.

## Materials and Methods

### Participants

R-fMRI data were acquired from 21 right-handed healthy adults (mean age  = 27.9 [SD 6.6] years; 9 males). All participants were college-educated native English speakers with no history of neurological or psychiatric disorders as confirmed by psychiatric interview, and had no contraindications to MRI. The study was approved by the institutional review boards of New York University (NYU) and the NYU School of Medicine. Prior written informed consent was obtained from all participants in accordance with the principles expressed in the Declaration of Helsinki. R-fMRI data from these participants have been reported in several published studies [32], [36]–[38], and are publically available for download at http://www.nitrc.org/frs/?group_id=274.

### CARE Questionnaire

All participants completed the Cognitive Appraisal of Risky Events (CARE) questionnaire [35]. The CARE is a self-report measure that assesses perceptions (“cognitive appraisals”) of the expected risk and expected benefit associated with a variety of risky behaviors (e.g., mixing drugs or alcohol, sex with a variety of partners, damaging/destroying public property), as well as the frequency of past involvement in those behaviors. The reliability and validity of this measure have been established across various adult and clinical populations [35]. Fromme and colleagues [35] reported that beliefs about the expected benefits associated with risky behaviors more reliably predicted engagement in those behaviors, relative to beliefs about the potential negative consequences. Given our goal of investigating the influence of real-world beliefs about risk-taking on functional connectivity in the brain, we limited our focus to participants' ratings on the expected benefit scale.

For the purposes of this study, participants' expected benefit scores were calculated as an average of their ratings on 4 factors: risky sexual behavior, heavy drinking, illicit drug use, and aggressive and illegal behaviors. Motivated conceptually by a desire to focus on those risky behaviors most likely to lead to adverse personal consequences, and following both previously published studies [39], [40] and the subsequently revised version of the CARE questionnaire (CARE-R) [41], 2 additional factors – irresponsible academic/work behaviors and high risk sports – were excluded.

We therefore obtained one score per participant that indicated an individual's level of expected benefit from engaging in a wide range of risky behaviors. Scores could range from 1 (not at all likely to experience positive consequences) to 7 (extremely likely to experience positive consequences). We interpreted lower expected benefit scores as indicating a more risk-averse personality, with higher expected benefit scores indicating a more risk seeking personality. These individual scores were entered as covariates of interest in group-level R-fMRI analyses to identify areas between which RSFC varied as a function of one's perceptions of the positive consequences associated with risk-taking.

### MRI Data Acquisition

All MRI scans were collected on a Siemens Allegra 3.0-T scanner at the NYU Center for Brain Imaging. Resting state fMRI scans consisted of 197 contiguous echo planar imaging whole-brain volumes (TR  = 2000 ms; TE  = 25 ms; flip angle  = 90°; 39 slices; matrix  = 64×64; field of view  = 192 mm; acquisition voxel size  = 3×3×3 mm^3^; duration  = 6.5 min). During each scan, participants were instructed to rest with eyes open while the word “Relax” was projected onto the center of the display screen. A high-resolution T1-weighted magnetization prepared gradient echo sequence (TR  = 2500 ms; TE  = 4.35 ms; TI  = 900 ms; flip angle  = 8°; 176 slices; field of view  = 256 mm) was also collected for spatial normalization and localization.

Twenty-one participants provided a resting state scan during a session in which the CARE questionnaire was also administered. We treated the scan collected during this session as our “*Primary Scan*” since it was collected closest in time to our measure of interest. Seventeen of these participants also provided a resting state scan (“Scan 1”) 4 to 16 months (mean  = 10.0 [SD 4.1]) prior to the Primary Scan (or “Scan 2”). Since we were interested in brain-behavior relationships that were stable across time, we treated Scan 1 as our “*Replication Scan,*” with which we investigated the stability of results emerging from the Primary Scan data analyses. Finally, 18 participants provided a third R-fMRI scan (“Scan 3”) collected ∼45 minutes after Scan 2, during the same session. In total, data from all 3 R-fMRI scans (Scans 1, 2 and 3) were available for 16 participants, data from two scans 5 months apart (Scans 1 and 2) for one participant, data from two scans 45 minutes apart (Scans 2 and 3) for two participants, and data from one scan only (Scan 2) for two participants.

### Image Preprocessing

Slice timing correction for interleaved acquisition (using Fourier-space time-series phase-shifting), motion correction (by aligning each volume to the eighth image using Fourier interpolation), and despiking (detection and reduction of extreme time series outliers) were carried out using Analysis of Functional NeuroImages (AFNI) [42]. Further preprocessing was performed using FMRIB Software Library (FSL; www.fmrib.ox.ac.uk) and included spatial smoothing using a Gaussian kernel (full width at half maximum  = 6 mm) and mean-based intensity normalization of all volumes by the same factor (i.e., all volumes were scaled by the same amount). Temporal bandpass filtering (0.009–0.1 Hz) and linear and quadratic detrending were then carried out in AFNI. Registration of each participant's high-resolution anatomical image to a common stereotaxic space (the Montreal Neurological Institute 152-brain template [MNI152]; 2×2×2 mm^3^ spatial resolution) was accomplished using a two-step process [43]. First, a 12-degrees-of-freedom linear affine transformation was estimated using *FLIRT* in FSL [44], [45], and then the registration was refined using nonlinear registration in FSL *FNIRT* [43]. This transformation was then applied to each participant's functional dataset.

### Nuisance Signal Regression

In order to control for the potential confounding effects of motion and physiological processes (e.g., cardiac and respiratory fluctuations), signals from 9 nuisance covariates were removed from each participant's preprocessed data. These were: white matter and cerebrospinal fluid signals (from masks generated using whole brain segmentation in FSL *FAST*), the mean global signal, and 6 motion parameters (i.e., X, Y, Z, pitch, yaw, and roll) as described in previously published studies [46], [47]. The resultant 4-D residual time series were used for subsequent participant-level analyses.

### Region of Interest and Seed Selection

A total of 12 regions of interest (ROIs) were selected as seed regions for RSFC analyses. Four of these regions were selected based on their association with the assessment of risk and reward (i.e., left and right OFC, left and right nucleus accumbens). Another six regions were selected based on their association with executive function and cognitive control (i.e., left and right IFG pars opercularis, left and right IFG pars triangularis, left and right middle frontal gyrus). All 10 of these seed ROIs were created using parcellation units derived from the Harvard-Oxford Cortical Structural Atlas probability thresholded at 75%. A high tissue probability was employed to maximize the likelihood of including only voxels that were classified as unique to each seed ROI. This conservative approach ensured that our atlas-based ROI definition minimized error due to normalization and smoothing across anatomical regions and across participants.

Two additional seeds located in the left and right dorsal anterior cingulate cortex (ACC) were also included. This region of ACC was previously shown to be functionally connected with a higher order cognitive network including lateral prefrontal regions implicated in cognitive control [48]. Given the functional heterogeneity of the ACC, spherical seeds (radius  = 4 mm; MNI x = −4/6, y = 34, z = 28) centered in this region were used as an alternative to the atlas-based approach.

### Participant-Level RSFC Analyses

Each participant's 4-D residuals volume was spatially normalized by applying the previously computed transformation to MNI152 2 mm standard space. We then extracted the mean time series from each seed by averaging across all voxels in each seed ROI. Using these mean time series, we performed a correlation analysis for each participant and each ROI using the AFNI program *3dfim+*, carried out in individuals' native space. This analysis produced participant-level correlation maps of all voxels in the brain that were positively and/or negatively correlated with the seed time series. Finally, these correlation maps were converted to Z-value maps using Fisher's *r*-to-*z* transformation. Participant-level correlation maps were computed for each scan separately (i.e., Scan 1, Scan 2, and Scan 3), and “Multi-Scan” mean correlation maps were created for participants with more than one scan. All maps were then transformed to MNI152 2 mm standard space.

Scripts containing a similar sequence of the processing commands employed here to compute seed-based RSFC have been released as part of the ‘1000 Functional Connectomes Project’ [49] (http://www.nitrc.org/projects/fcon_1000).

### Group-Level RSFC Analyses

In our Primary Scan analyses, for each seed ROI, group-level analyses were carried out using a mixed-effects model implemented in FSL *flameo* (ordinary least squares). In addition to the group mean vector, demeaned expected benefit scores and two nuisance variables (demeaned age and sex) were included in the model. Cluster-based statistical correction for multiple comparisons was performed using Gaussian random field theory (Z>2.3; cluster significance: *p*<0.008 corrected; *p*<0.008 was selected to take into account the number of independent seed regions employed [0.008 = 0.05/6]. Six, as opposed to 12, seed ROIs were considered given the high degree of correlated activity between homotopic seed regions [50], [51]). This group-level analysis produced two types of thresholded Z-statistic maps: 1) voxels exhibiting significant positive and negative correlation with each seed ROI, and 2) voxels whose correlation with the seed ROI exhibited significant variation in association with the expected benefit scores (i.e., regions in which connectivity with the seed region was predicted by the level of benefit participants expected from engaging in risky behaviors).

### Consistent RSFC Relationships with Expected Benefit

Our main aim was to identify brain-behavior relationships that were stable across time. Specifically, we sought to identify regions whose functional connectivity with a seed ROI was significantly associated with expected benefit scores across multiple scans.

We used our Primary Scan (Scan 2) to identify regions whose connectivity with a seed ROI was significantly predicted by expected benefit scores. We then tested for the presence of the same relationship in our Replication Scan (Scan 1) by extracting the mean correlation (with the same seed ROI) for significant clusters identified using the Primary Scan, and correlating the resultant values with expected benefit scores. Scan 2 was chosen as the Primary, or “base,” scan because its acquisition was closest in time to the collection of the questionnaire. This allowed us to investigate whether significant relationships between expected benefit and RSFC observed in the Primary Scan (at the time the CARE was administered) were also observed in the Replication Scan (collected 4 to 16 months earlier). Given the presence of *a priori* regions of interest and predicted findings based upon the Primary Scan analyses, statistical correction was not necessitated nor employed in the Replication Scan analyses [52], [53]. Correlations were computed both before and after controlling for the effects of age and sex.

### Group Differences in RSFC for Lower vs. Higher Expected Benefit Scores

To further investigate the relationship between RSFC and expected benefit (EB) scores, participants were divided into two groups (i.e., Lower EB and Higher EB) based on a median split. Twenty participants were included – participants with the 10 lowest scores were included in the Lower EB group, and participants with the 10 highest scores were included in the Higher EB group. One participant with intermediate scores was excluded. For each seed ROI whose significant relationships with expected benefit replicated across Scans 1 and 2 (e.g. right IFG pars opercularis and left nucleus accumbens, see Results below), a two-sample t-test was carried out on participants' Multi-Scan mean correlation maps (i.e., maps of RSFC averaged across all available scans per participant) using *FEAT* in FSL. In addition to the two group mean vectors, two nuisance variables per group (age and sex, demeaned separately for each group) were included in the model. Cluster-based statistical correction for multiple comparisons was performed using Gaussian random field theory (Z>2.3; cluster significance: *p*<0.025 corrected, taking into account the number of seed ROIs of interest [0.025 = 0.05/2]). This group-level analysis produced thresholded Z-statistic maps comparing the functional connectivity of each seed ROI between groups (i.e., Lower EB>Higher EB and Higher EB>Lower EB) as well as maps of the significant positive and negative functional connectivity of each seed ROI within each group (i.e., Lower EB positive RSFC, Lower EB negative RSFC, Higher EB positive RSFC, Higher EB negative RSFC). These analyses allowed us to determine whether the same significant relationships between RSFC and expected benefit observed in the previously described covariate analyses could also be observed in a direct comparison of the RSFC maps between participants who expected more or less benefit from engaging in risky behaviors. As such, these group comparisons are a replication of the correlation analyses and have been included to provide a complementary, alternative view (not an independent test) of the observed brain-behavior relationships.

## Results

### Behavioral Results

Expected benefit ratings from participants in the current study ranged from 1.00 to 4.57 on a 7-point Likert scale (mean = 2.54 [SD 0.88]). Although this mean score suggests that, on average, participants did not expect positive consequences from engaging in risky behaviors, across participants there was sufficient range in the ratings to provide an estimate of the effect of expected benefit on RSFC in subsequent neuroimaging analyses.

### Neuroimaging Results

#### Consistent RSFC Relationships with Expected Benefit

Significant relationships between expected benefit scores and RSFC were detected for several seed regions (for full results from the Primary Scan, see Table S1). Two of these relationships were stable across scans carried out 4 to 16 months apart (i.e., Primary and Replication Scans). Specifically, we observed a stable negative association between expected benefit scores and RSFC between 1) the IFG and anterior insula, and 2) the nucleus accumbens and parieto-occipital cortex.

**Figure 1:**
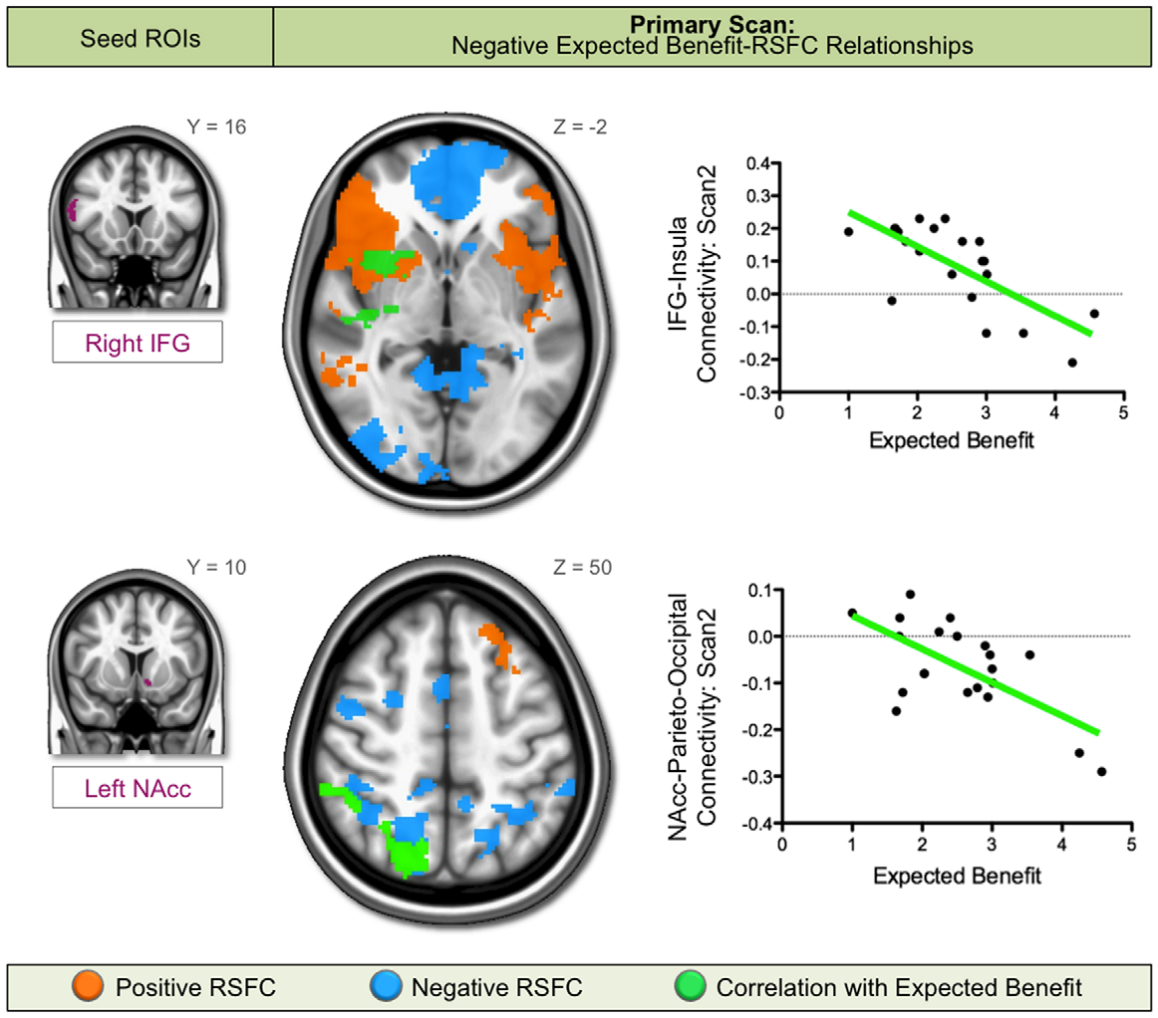
Negative relationships between expected benefit and RSFC from the Primary Scan analyses. A negative relationship with expected benefit was observed in the Primary Scan (Scan 2) for resting state functional connectivity (RSFC) between the right inferior frontal gyrus (IFG) pars opercularis seed and right insula (top row), and the left nucleus accumbens (NAcc) seed and right parieto-occipital cortex (bottom row). These negative relationships were observed as either decreasing positive connectivity (green overlaid on red-orange) or increasing negative connectivity (green overlaid on light blue) in the voxel-wise correlation maps (middle column). These relationships are also illustrated in the scatter plots comparing RSFC values and expected benefit scores (right column).

**Figure 2:**
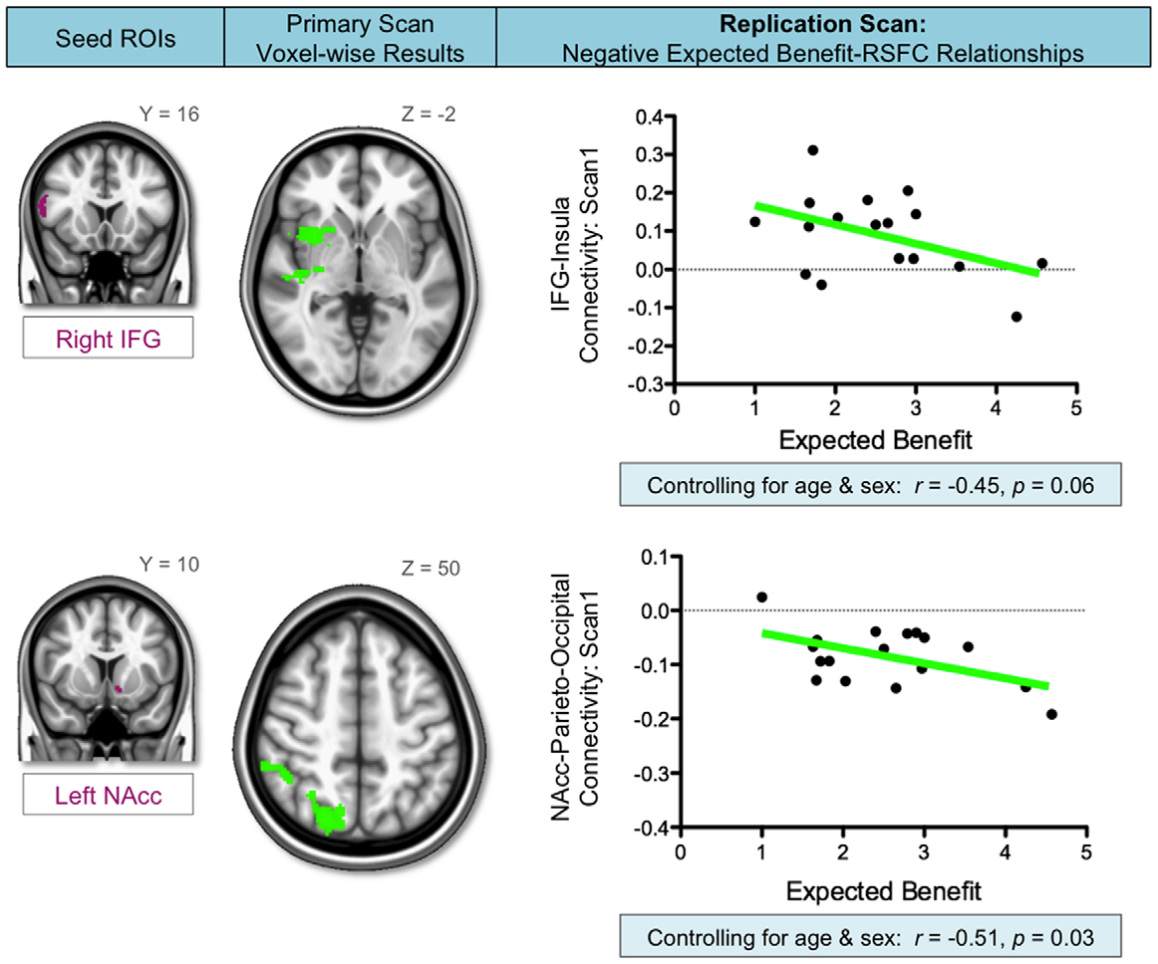
Negative relationships between expected benefit and RSFC from the Replication Scan analyses. A negative relationship with expected benefit was also observed in the Replication Scan (Scan 1) for resting state functional connectivity (RSFC) between the right inferior frontal gyrus (IFG) pars opercularis seed and right insula cluster that was significant in the Primary Scan (top row), and the left nucleus accumbens (NAcc) seed and right parieto-occipital cortex cluster that was significant in the Primary Scan (bottom row). These relationships replicate those observed in the Primary Scan analyses, and are illustrated in the scatter plots (right column).

Increased expected benefit from engaging in risky behaviors was associated with weaker positive connectivity between the IFG and insula. In other words, the less benefit expected from risk-taking (i.e., the more risk-averse participants were), the stronger the positive connectivity between IFG and insula. In the Primary Scan, we observed a significant voxel-wise inverse relationship between expected benefit and RSFC between the right IFG pars opercularis seed and right insular cortex (Fig. 1, top row). To test for stability across time, RSFC values between the right IFG pars opercularis seed and the insula cluster exhibiting the significant relationship in the Primary Scan were extracted from the Replication Scan. Using these Replication Scan values, the same significant inverse correlation with participants' expected benefit scores was observed, *r*(17)  = −0.49, *p*<0.05 (Fig. 2, top row). This relationship just escaped significance when controlling for age and sex, *r*(17)  = −0.45, *p* = 0.06.

Increased expected benefit from engaging in risky behaviors was associated with increased negative connectivity between left nucleus accumbens and parieto-occipital cortex. In other words, the less participants expected to benefit from risk-taking (more risk-averse), the weaker their negative connectivity between nucleus accumbens and parieto-occipital cortex. In the Primary Scan, we observed a significant voxel-wise inverse relationship between expected benefit scores and RSFC between the left nucleus accumbens seed and right parieto-occipital cortex (e.g., lateral occipital cortex, supramarginal and angular gyri, superior parietal lobule, precuneus) (Fig. 1, bottom row). As before, to test for stability across time, RSFC values between the left nucleus accumbens seed and the parieto-occipital cluster exhibiting the significant relationship in the Primary Scan were extracted from the Replication Scan. Using these Replication Scan values, an inverse correlation with participants' expected benefit scores was observed, which was marginally significant when examined alone, *r*(17)  = −0.41, *p* = 0.09, but was significant after controlling for age and sex, *r*(17)  = −0.51, *p*<0.05 (Fig. 2, bottom row).

#### Group Differences in RSFC for Lower vs. Higher Expected Benefit Scores

To further understand the associations between expected benefit and RSFC, we conducted a group analysis in which we compared RSFC between participants who reported lower and those who reported higher expected benefit from engaging in risky behaviors. For these analyses, we calculated mean (Multi-Scan) RSFC maps for each participant, and focused on those seeds whose RSFC patterns were consistently negatively associated with expected benefit scores across scans (i.e., right IFG pars opercularis and left nucleus accumbens).

**Figure 3:**
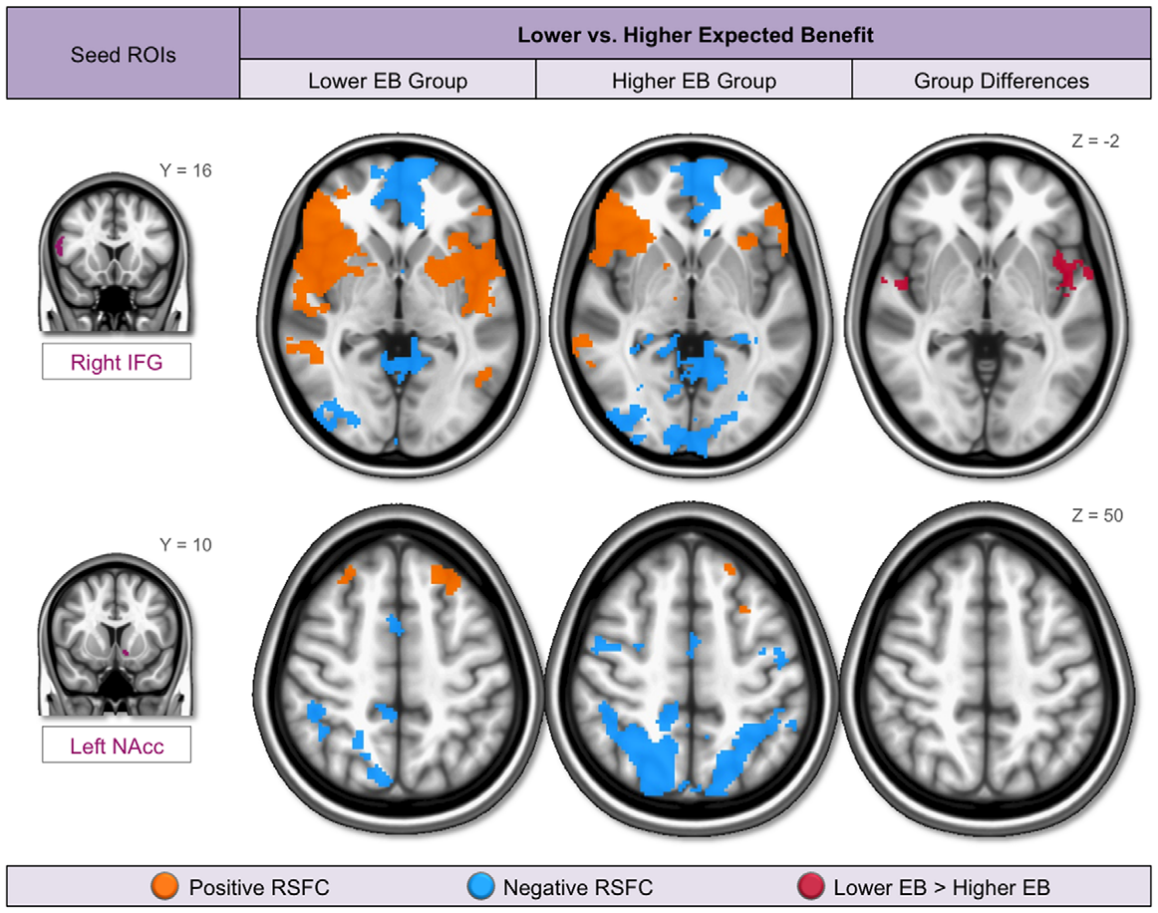
Group differences in RSFC for participants with lower vs. higher expected benefit ratings. Resting state functional connectivity (RSFC) for participants reporting Lower expected benefit (EB) from engaging in risky behaviors, Higher EB, and group differences (2^nd^, 3^rd^, and 4^th^ columns, respectively). Significantly greater positive functional connectivity was observed between the right inferior frontal gyrus (IFG) pars opercularis seed and bilateral insula in the Lower > Higher EB group contrast (top row, 4^th^ column). No significant group differences in RSFC were observed for the left nucleus accumbens (NAcc) seed (bottom row, 4^th^ column), but patterns of negative functional connectivity were suggestive of a Higher > Lower EB trend in right parieto-occipital cortex (bottom row, 2^nd^ and 3^rd^ columns). Both of these findings are consistent with results from the previous covariate analyses.

Participants in the Lower EB group (mean age = 30.4 [SD 8.5]; 5 males) had an average expected benefit score of 1.82 (SD 0.39), while participants in the Higher EB group (mean age = 25.5 [SD 3.3]; 4 males) had an average score of 3.26 (SD 0.65). In order to account for any differences between groups, age and sex were entered as nuisance covariates in the analysis. As expected, direct comparisons between groups revealed significantly greater positive functional connectivity between the right IFG pars opercularis seed and bilateral insular cortex in the Lower EB, relative to the Higher EB, group (Fig. 3, top row, 4th column). As can be seen in Figure 3 (top row, 2^nd^ and 3^rd^ columns), regions of insula positively correlated with the IFG were more extensive in the Lower than the Higher EB group. This finding was consistent with the results of the expected benefit covariate analysis, demonstrating stronger positive functional connectivity between these regions with lower expected benefit scores.

In contrast, no significant group differences in RSFC were observed for the left nucleus accumbens seed (Fig. 3, bottom row, 4^th^ column). However, as illustrated in Figure 3 (bottom row, 2^nd^ and 3^rd^ columns), the region of parieto-occipital cortex whose activity was negatively correlated with nucleus accumbens was more extensive in the Higher than the Lower EB group. Although the Higher and Lower EB groups did not differ significantly in RSFC, the overall patterns of RSFC were consistent with the finding of weaker negative functional connectivity with decreased expected benefit scores (or stronger negative functional connectivity with increased expected benefit scores) observed in previous analyses.

## Discussion

Individual differences in beliefs about the consequences of engaging in risky behaviors are reflected in the brain's intrinsic functional architecture. The intrinsic functional connectivity between brain regions typically engaged by risky decision-making tasks was associated with the extent to which a participant endorsed positive outcomes of risky behavior. Not only were these brain-behavior relationships observed in the absence of an explicit task, they were stable across a time interval of approximately one year. Our findings emphasize the utility of RSFC methods for the investigation of inter-individual differences in brain function associated with enduring behavioral traits and tendencies (e.g., risk-aversion). Moreover, they demonstrate that information about individuals' beliefs about the consequences of real-world risk-taking can elicit predictable brain-behavior relationships without the requirement to perform a specific task (e.g., gambling for money), which necessarily limits the type of risky behavior interrogated.

### Relationship between Expected Benefit and RSFC of Brain Regions Implicated in Control and Risk-Aversion

We observed a stable (over 4–16 months) brain-behavior relationship for regions typically implicated in cognitive control and risk-aversion. Specifically, decreased expected benefit from risky behavior (interpreted as reflecting a more risk-averse personality style) was associated with stronger positive intrinsic connectivity between the IFG and anterior insula. A group comparison further demonstrated increased insular participation in the IFG intrinsic connectivity network in individuals endorsing lower relative to higher expected benefit from risky behavior. These observations are congruent with the consistent implication of these two brain regions in risky decision-making, most notably when individuals respond in a more risk-averse manner.

In the context of cognitive or behavioral control, the IFG has been repeatedly implicated in inhibitory control [54]–[56]. Abnormal activation in this area has been associated with the behavioral dysregulation characteristic of Attention-Deficit/Hyperactivity Disorder (ADHD) [57], as well as substance abuse disorders [58], [59]. In gambling tasks, IFG activation has been observed to track risk prediction, increase in response to lower-risk options, and correlate with individuals' behavioral risk-aversion [13], [60].

Perhaps due to its implication in addictive disorders [61], the insula has recently become an area of increased focus in neuroimaging studies of risky decision-making [62]. Increased insular activation has been associated with the perceived risk and risk-prediction of a choice [60], [63], and with risk-free compared to risky choices [21]. The anterior insula specifically has been implicated in adopting a loss-minimizing decision strategy [64] and in learning to avoid losses [65]. Consistent with this pattern, a recent meta-analysis determined that the anterior insula was particularly involved in risk-related processing when there was the potential for loss [66].

Disruption of insula function via lesions [62], [67] or sleep deprivation [68] has been associated with disadvantageous decision-making under risk. This effect has been suggested to reflect a disruption in signaling the probability of aversive outcomes [67], and a diminished response to losses [68]. Conversely, Paulus et al. [14] reported that increased insula activation during risky decision-making was associated with individuals' degree of harm avoidance and neuroticism. In fact, anxiety-prone individuals, who are typically extremely risk-averse, exhibit disproportionately increased insula activation when processing salient, emotional stimuli [69]. This altered insula responsivity has been proposed as a key neural mechanism underlying one's susceptibility to anxiety disorders [70].

Our findings draw further attention to a fundamental role for both the IFG and insula in the cognitive evaluation of risk. Functional interactions between these two brain regions may contribute to the avoidance of risky behaviors.

### Relationship between Expected Benefit and RSFC of Brain Regions Implicated in Reward and Risky Decision-Making

We observed a second stable brain-behavior relationship for the nucleus accumbens, a region long implicated in reward and risky decision-making. Decreased expected benefit from risky behavior (i.e., a more risk-averse personality style) was associated with weaker negative intrinsic connectivity between the left nucleus accumbens and right parieto-occipital cortex. In general, positive and negative functional connectivity provide different types of information about the brain's intrinsic functional organization. Specifically, negative functional connectivity is most often observed between brain regions that are members of **different** functional networks (e.g., the “task positive” and “task negative” networks [71]), while positive functional connectivity is typically observed between brain regions that are members of the **same** network (e.g., posterior cingulate and ventromedial prefrontal cortices, which are nodes within the “task negative” network). Although there is controversy regarding the interpretation of negative RSFC [72], our observation of a negative relationship between expected benefit scores and (negative) RSFC between the nucleus accumbens and parietal cortex suggests that their respective functional networks are less segregated (or differentiated) from each other with decreased risk seeking/increased risk-aversion. In other words, we observed an association between the evaluation of risk and the strength of the intrinsic relationship between two *networks*, one based in the nucleus accumbens and the other based in parietal cortex. This finding differs from our findings for the IFG and insula, where the cognitive evaluation of risk was associated with the *spatial extent* of the IFG-based functional connectivity network (which included the insula).

The nucleus accumbens is commonly implicated in the processing of risk and reward. Increased activation in the ventral striatum, including the nucleus accumbens, has been associated with risk seeking decisions [21], increased probability of risky choices [13], and calculation of reward prediction error [60]. Altered activation in the nucleus accumbens as a result of sleep deprivation [68] has been associated with disadvantageous decision-making. In clinical disorders such as ADHD, commonly associated with decision-making impairments [73], hypoactivation of the nucleus accumbens has been observed during reward processing tasks [74]–[76].

Although perhaps less commonly discussed, activation in regions of lateral parieto-occipital cortex (e.g., superior parietal lobule) has been reported in several studies of risky decision-making. Right parietal activation has been associated with risk-related processing especially in situations involving choice [66] and with the use of a gain-maximizing decision strategy [64]. Lateral parietal activation during risky compared to safe decisions has also been associated with increased measures of impulsiveness [77].

With regard to interactions between nucleus accumbens and parietal cortex, co-activation of the ventral striatum and right inferior parietal cortex has been associated with loss aversion. Loss-related decreases in activation in these areas were greater than gain-related increases in activation. This differential neural response to loss predicted individual differences in behavioral loss aversion during risky decision-making [78]. While the functional networks of the nucleus accumbens and parietal cortex may make differential contributions to risk-related cognitions and behavior, interactions between them may contribute to risk-aversion. This suggestion is consistent with our observation of weaker separation between these networks in participants who were more risk-averse, relative to participants who were more risk seeking.

### The Brain's Intrinsic Functional Architecture Sets the Stage for Behavior

Our findings add to a growing literature demonstrating that individual differences in behavior or behavioral tendencies are reflected in the brain's intrinsic functional architecture [32], [79], [80]. Specifically, we observed that individual differences in risk-related behavioral tendencies were associated with variability in positive functional connectivity between the IFG and insula (members of the same resting state network), and variability in negative functional connectivity between the nucleus accumbens and parietal cortex (members of different networks).

Beyond brain-behavior relationships, previous work has also shown that individual differences in RSFC predict task-evoked fMRI activation [81]. Interestingly, those relationships were most robust for regions of variable RSFC, located at the edges of, or between networks [32], [81]. This suggests that, while there is a shared “core” intrinsic functional architecture [49] that presumably supports those aspects of cognition and behavior that are common to all humans, there is also considerable variation in that architecture, and it is this variation that underlies individual differences. In other words, while the core nodes of functionally connected networks tend to show robust, stable patterns of RSFC across individuals, regions that are more variable across individuals are those that provide us with information about individual differences in brain-behavior relationships. Characterizing these relationships in the context of intrinsic functional circuits can provide a better understanding of the concerted activation among brain regions and differences among individuals during task performance. This relationship between the organization of the brain's functional architecture and behavioral tendencies/personality traits may provide a window into both normal variation and pathological extremes of cognition and behavior.

### Limitations

We used the CARE questionnaire to quantify participants' beliefs about the consequences of engaging in risky behavior. This required the assumption that individuals' ratings of the expected benefit from risky behavior were indicative of their level of risk seeking (higher ratings) or risk-aversion (lower ratings). Our use of the CARE questionnaire was motivated by a desire to capture individual differences in risk-related personality traits associated with real-world risk-taking behaviors. Fromme and colleagues [35] reported that beliefs about positive consequences (expected benefit) were more predictive of actual risk-taking than beliefs about negative consequences. It is important to point out that, though our participants reported a range of expected benefit scores, no one endorsed extremely high ratings. As such, our discussion of “higher” scores does not imply extreme levels of risk seeking, but suggests that these individuals may be more prone to risk-taking, or less risk-averse, than others. Future studies should employ a range of risk-related personality measures and behavioral tasks in order to more fully characterize these brain-behavior relationships.

Even though beliefs about the potential benefits of engaging in risky behaviors have been shown to be more predictive of actual risk-taking than beliefs about their negative consequences, it could be argued that our participants' expected benefit scores reflect a preoccupation with the positive, and an under-appreciation of the negative consequences of these behaviors. Though not a central focus of the brain-behavior relationships examined in the current study, in order to address this consideration, we examined participants' ratings of expected risk (i.e., their ratings of the likelihood of experiencing negative consequences as a result of risky behavior) in relation to their ratings of expected benefit. For all 4 dimensions of risky behavior included in our analyses, participants indicated that the likelihood of negative consequences was greater than the likelihood of positive consequences (Negative-to-Positive Ratio: risky sexual behavior  = 2.30; heavy drinking  = 1.18; illicit drug use  = 1.92; aggressive and illegal behaviors  = 3.15). This pattern of results suggests that participants were well aware of the potential negative consequences associated with these behaviors, and that our results, although focused on ratings of expected benefit, are not merely due to an undervaluation of possible adverse outcomes.

One critical aspect of the current study was the identification of brain-behavior relationships that were stable across time. This limited our focus to those results that remained significant across scans occurring roughly one year apart. Since the CARE questionnaire was not administered at both time points, we cannot be sure that participants' beliefs about risk-taking did not change in the time between scans. This could be one possible explanation for why we did not observe stable relationships between expected benefit and RSFC in several of our *a priori* regions of interest. This lack of stability could reflect a number of factors such as limited power, variable fMRI signal, or signal loss. However, an examination of the group-level masks for each analysis indicated good signal in all seed ROIs for all scans, ruling out signal loss as an explanation. Further, the demonstration of stable relationships for the IFG/insula and nucleus accumbens/parietal cortex mitigates concerns about signal variability. Another explanation is that relationships observed for areas such as the middle frontal gyrus reflect transient, state effects, rather than enduring behavioral tendencies. Activation in some of these regions may be most reliably elicited during the evaluation of immediate risks and rewards. These possibilities merit further study with both task-based and resting state approaches.

### Implications and Future Directions

If replicated, results from the current study will have implications for the understanding, characterization, and possible early identification of abnormal functional connectivity between brain regions associated with risk-related processing. It is well established that a wide range of psychiatric disorders are associated with abnormal behavior and neural activity during risky decision-making [2], [7], [82]. Our results suggest that RSFC methods can identify variations in patterns of intrinsic connectivity between brain regions as a function of individuals' level of risk seeking or risk-aversion. Future studies will pursue these brain-behavior relationships in populations known to engage in risky behaviors (e.g., substance abuse), as well as populations characterized by extreme risk-aversion (e.g., anxiety disorders). In this way, we can characterize the relationship between intrinsic brain connectivity and both normal and pathological levels of risk seeking and risk-aversion. Though initial demonstrations have been promising [83], [84], considerable work is required in order to demonstrate the specificity and sensitivity of any putative RSFC biomarkers of psychiatric disorders (e.g., ROC curves [85]). Nonetheless, a better understanding of these brain-behavior relationships can contribute to the development of potential diagnostic markers of an increased risk for maladaptive decision-making, and to possible early intervention strategies.

## Supporting Information

Table S1 Regions exhibiting a significant relationship between expected benefit from engaging in risky behaviors and resting state functional connectivity in the Primary Scan.
(0.04 MB DOC)
