# Supplementary material for: Your Resting Brain CAREs about Your Risky Behavior
Source: PLoS One. 2010 Aug 19;5(8):e12296. doi: 10.1371/journal.pone.0012296 (PMC2924392; doi:10.1371/journal.pone.0012296)
Supplement: Table S1 — Regions exhibiting a significant relationship between expected benefit from engaging in risky behaviors and resting state functional connectivity in the Primary Scan. (0.04 MB DOC) [file pone.0012296.s001.doc]

Table S1. Regions exhibiting a significant relationship between expected benefit from engaging in risky behaviors and resting state functional connectivity in the Primary Scan.

|  |  | Positive Correlation with EB | | | Negative Correlation with EB | | |
| --- | --- | --- | --- | --- | --- | --- | --- |
| Seed region | Connected region(s) | # of voxels | Peak Z | Peak Coordinates | # of voxels | Peak Z | Peak Coordinates |
| Left IFG Opercularis | Left Precentral Gyrus (Premotor)/ Superior Parietal Lobule |  |  |  | 1486 | 4.56 | -6, -16, 72 |
| Right IFG Opercularis | Right Insula/ Putamen |  |  |  | 789 | 4.04 | 34, 6, -2 |
| Left Middle Frontal Gyrus | Right Occipital Pole/ Lateral Occipital Cortex |  |  |  | 577 | 3.82 | 20, -94 4 |
| Right Middle Frontal Gyrus | Right Occipital Pole/ Lateral Occipital Cortex |  |  |  | 756 | 4.37 | 16, -92, 10 |
| Left Nucleus Accumbens | Right Lateral Occipital Cortex/ Supramarginal Gyrus/ Angular Gyrus/ Superior Parietal Lobule/ Precuneus (IPL, IPS) |  |  |  | 966 | 4.38 | 28, -68, 46 |
| Right Nucleus Accumbens | Bilateral Lingual Gyrus/ Intracalcarine Cortex/ Occipital Pole | 1262 | 4.76 | 2, -84, -6 |  |  |  |

Abbreviations: EB – expected benefit; IFG – inferior frontal gyrus; IPL – inferior parietal lobule; IPS – intraparietal sulcus. Coordinates are reported in MNI152 space.
